# Urinary Risk Profile, Impact of Diet, and Risk of Calcium Oxalate Urolithiasis in Idiopathic Uric Acid Stone Disease

**DOI:** 10.3390/nu15030572

**Published:** 2023-01-21

**Authors:** Roswitha Siener, Patricia Löhr, Albrecht Hesse

**Affiliations:** University Stone Center, Department of Urology, University Hospital Bonn, 53127 Bonn, Germany

**Keywords:** kidney stones, urolithiasis, nephrolithiasis, urinary pH, uric acid, calcium, oxalate, diet, overweight, obesity

## Abstract

The role of diet in the pathogenesis of uric acid (UA) nephrolithiasis is incompletely understood. This study investigated the effect of dietary intervention on the risk of UA stone formation under standardized conditions. Twenty patients with idiopathic UA stone disease were included in the study. Dietary intake and 24 h urinary parameters were collected on the usual diet of the patients and a standardized balanced mixed diet. Although urinary UA excretion did not change, the relative supersaturation of UA decreased significantly by 47% under the balanced diet primarily due to the significant increase in urine volume and pH. Urinary pH was below 5.8 in 85% of patients under the usual diet, and in 60% of patients under the balanced diet. The supersaturation of calcium oxalate declined significantly under the balanced diet due to the significant decrease in urinary calcium and oxalate excretion and the increase in urine volume. Dietary intervention is a key component in the management of UA nephrolithiasis. Urinary calcium and oxalate excretion should also be monitored in patients with pure UA calculi to reduce the risk of mixed stone formation with calcium oxalate. Lower urinary pH in UA stone patients can only be partially explained by diet.

## 1. Introduction

Uric acid (UA) stones account for about 8% of all urinary calculi [1,2,3]. UA stones are about four times more common in men than in women [1,3]. The proportion of UA calculi increases strongly in both genders after approximately the age of 50 years [1,2,3]. UA is present in two different crystal forms, anhydrous uric acid and uric acid dihydrate [3]. UA stones occur predominantly as pure UA or as a mixture with calcium oxalate [2]. UA stone patients are considered to be at high risk of stone recurrence [4]. Moreover, an association between UA urolithiasis and a higher prevalence of chronic kidney disease has been observed [5].

Characteristic urinary abnormalities in UA stone disease include persistently acidic urinary pH, hyperuricosuria, and low urine volume [6,7,8,9]. An unduly low urinary pH is the major pathogenetic factor for the crystallization of poorly soluble undissociated UA, increasing the propensity for UA stone formation [6,9,10]. Hyperuricosuria may result from elevated production of UA, which may be mainly due to a higher dietary purine intake [6,11]. UA can be the only constituent of the stone, but may also promote mixed calcium oxalate and UA stone formation through heterogeneous nucleation and epitaxial crystal growth [12,13,14]. Low urine volume additionally contributes to UA stone formation by increasing urinary concentration and supersaturation of UA. UA nephrolithiasis has been associated with several components of the metabolic syndrome including abdominal obesity, type 2 diabetes, and dyslipidemia [15,16,17,18,19,20].

Due to the high risk of stone recurrence, personalized therapy is of utmost importance in the management of UA stone disease. Dietary factors may affect the urine composition of patients with UA nephrolithiasis. Based on metabolic evaluation and nutritional assessment, dietary treatment should be tailored to the specific metabolic abnormalities and comorbidities of the individual patient. However, information on the dietary patterns of patients with UA calculi is limited. In particular, to our knowledge, studies on the effect of a controlled standardized dietary intervention on the urinary risk profile of UA stone formers are lacking. Therefore, the aim of the present study was to determine urinary abnormalities of UA stone formers under standardized metabolic conditions to exclude exogenous determinants of urinary risk factors for stone formation, especially diet. In addition, we compared urinary composition under the usual, self-selected diet and under the controlled, standardized balanced diet to identify the impact of diet in idiopathic UA stone disease.

## 2. Materials and Methods

### 2.1. Patients

A total of 20 patients (17 men and 3 women) with UA urolithiasis were included in this study. The patients were admitted to the University Stone Center at the Department of Urology of the University Hospital Bonn for an inpatient metabolic evaluation under standardized conditions. Inclusion criterion was stone analysis of a recent stone episode with at least 50% anhydrous uric acid and/or uric acid dihydrate as stone component. Stone analysis was performed by Fourier transform infrared spectroscopy. Patients did not receive dietary counseling prior to participation in the study and were asked to maintain their usual dietary habits before study entry. Patients did not receive any medication that could affect UA, oxalate and calcium metabolism or acid-base status, such as alkali citrate, allopurinol, or thiazides, for 4 weeks before study entry and during the study. The study was approved by the Ethics Committee of the Medical Faculty of the University of Bonn (430/19) and informed consent was obtained from each patient.

### 2.2. Study Procedure

Medical history, dietary records, anthropometric, clinical, and 24 h urinary parameters were obtained from all patients at baseline under their usual, self-selected diet. Dietary intake of patients under their habitual diet was recorded using a 7-day food record. Patients provided a detailed description of the types and weighed amounts of all foods consumed. The nutrient composition of the foods was calculated using the PRODI 5.3 computer program (Nutri-Science GmbH, Freiburg, Germany). The oxalate content of the foods measured in our laboratory was entered into the database [21,22,23]. Sodium intake was estimated from 24 h urinary sodium excretion.

In the following phase, the patients were maintained on a balanced mixed standardized diet for 11 days [7]. After a few days of adaptation, this standardized diet, i.e., consistent daily intake of the prescribed foods and fluids, leads to a metabolic steady state, so that constant urinary values are achieved [7]. Fluid intake through beverages was 2.5 L per day. Patients collected 24 h urines during their self-selected diets and after 7 days on the standardized balanced diet. Gastrointestinal oxalate absorption of the patients was measured using the standardized [^13^C_2_]oxalate absorption test [24]. The [^13^C_2_]oxalate absorption test was conducted on days 9 and 10 under controlled, standardized conditions.

### 2.3. [^13^C_2_] Oxalate Absorption Test

The [^13^C_2_]oxalate absorption test was performed over a period of two consecutive days under a standardized diet [24]. Patients ingested 50 mg sodium [^13^C_2_]oxalate, equivalent to 33.8 mg [^13^C_2_]oxalic acid, on the morning of the second day in a fasting state and collected fractional 24 h urines. Unlabeled and labeled oxalate was quantified by gas chromatography–mass spectrometry. Absorption is expressed as a percentage of the labeled oxalate dose. Intestinal oxalate absorption exceeding 10% is defined as hyperabsorption [7,25].

### 2.4. Urinary Parameters

Urine volume, density, pH, and concentrations of sodium, calcium and potassium (ion selective electrode), magnesium (xylidyl-blue reaction), chloride (coulomb metric titration), inorganic phosphate (phosphate molybdate reaction), inorganic sulfate (nephelometry), ammonium (ion selective electrode), uric acid (enzymatically, uricase), citrate (enzymatically, citrate lyase), oxalate (enzymatically, oxalate oxidase), and creatinine (Jaffé reaction) were measured. The ion activity products (AP) of uric acid, calcium oxalate, struvite, and brushite were determined according to Tiselius [7,26,27]. The risk of stone formation, computed as relative supersaturation of uric acid, calcium oxalate, struvite, and brushite, was calculated using the computer program EQUIL2 (University of Florida, Gainesville, FL, USA) [28]. Laboratory quality certification was available for each parameter.

### 2.5. Statistical Analysis

The nonparametric Wilcoxon test was used to compare parameters before and after dietary intervention. The association between continuous variables was assessed using Spearman’s rank correlation. Results are expressed as mean ± standard deviation. The significance level was considered as *p* < 0.05. All statistical tests were two-tailed. Statistical analysis was performed with SPSS^®^ for Windows version 27.0 (IBM, Armonk, New York, NY, USA).

## 3. Results

### 3.1. Patient Characteristics

Twenty patients, 17 men and three women, with idiopathic UA urolithiasis were included in the study. Patient characteristics are shown in Table 1. The mean age of patients was 55.2 ± 12.8 years (range: 32–76 years). In total, 89% of all patients were overweight or obese. The two patients with normal BMI were females. One of nine male UA stone patients who underwent the [^13^C_2_]oxalate absorption test was diagnosed with intestinal hyperabsorption greater than 10%. Hypertension was present in 50% and type 2 diabetes in 20% of the patients. Hyperuricemia, defined as serum UA > 6.4 mg/dL, was diagnosed in 40% of patients. Mean serum uric acid concentration was 6.2 ± 1.4 mg/dL on the usual, self-selected diet of UA stone patients and 6.4 ± 1.7 mg/dL on the standardized balanced mixed diet (*p* = 0.410). A family history of stones was documented in one third of the patients. In 85% of patients, stone analysis revealed 100% UA, with varying proportions of anhydrous uric acid and uric acid dihydrate. The urinary stones of the remaining three patients were admixed with whewellite in proportions of 5%, 15%, and 50%.

### 3.2. Urine Composition

The urinary risk profile of UA stone patients on their usual, self-selected diets and the balanced mixed standardized diet is shown in Table 2. The relative supersaturation of UA decreased significantly by 47% during the intake of the balanced mixed diet, although urinary UA excretion did not change significantly. The relative supersaturation of calcium oxalate declined significantly and to the same extent. Moreover, the ion activity products of UA, calcium oxalate, and brushite were significantly lower on the balanced mixed diet than on the usual, self-selected diet of UA stone patients. Urine volume and pH increased significantly, while urine density, calcium, oxalate, sodium, chloride, phosphate, and sulfate excretion declined significantly during the intake of the balanced mixed diet. No change was observed in the other urinary parameters.

A urinary pH below 5.8 was the most common urinary abnormality on the habitual diet (Table 3). On the usual, self-selected diet, urinary pH was below 5.40 in 40% of patients and between 5.40 and 5.79 in 45% of patients. On the balanced mixed standardized diet, urinary pH was still below 5.8 in 60% of patients. Although the mean urine volume increased significantly during the intake of the balanced diet, the urine volume was ≥2.0 L/24 h in only 65% of the stone formers. Urinary UA excretion ≥4.0 mmol/24 h was diagnosed in 40% of UA stone patients on their usual diet, but only in 15% on the balanced mixed diet. While hyperoxaluria was present in 25% of UA stone formers on the usual, self-selected diet, none of the patient had hyperoxaluria on the balanced mixed diet.

No correlation was observed between urinary pH and body weight (usual diet: R = −0.201, *p* = 0.410; balanced diet: R = −0.126, *p* = 0.609) and between urinary pH and BMI (usual diet: R = −0.291, *p* = 0.226; balanced diet: R = 0.025, *p* = 0.920) under either diet.

### 3.3. Nutrient Intake

Nutrient intake under the usual diet and the balanced mixed diet is depicted in Table 4. While the mean daily intakes of protein, fat, methionine, cystine, cholesterol, purines, and sodium were significantly lower, the mean daily intakes of carbohydrates, fiber, potassium, and water (from food and beverages) were significantly higher with the balanced mixed standardized diet. During the controlled, standardized balanced diet, the consumption of alcohol was not allowed.

## 4. Discussion

The classic triad of UA nephrolithiasis includes low urine volume, acidic urinary pH, and hyperuricosuria [6,7,8,9]. Several diet-independent factors have been hypothesized to be involved in UA stone formation through their effects on urine composition. The demographic characteristics that have been associated with UA stone disease are age and gender. UA calculi are more common in men than women, and occur more frequently at older ages [1,2,3]. The present study confirmed a higher mean age and a male preponderance in UA stone patients. In a study of kidney stone patients, the gender disparity was attributed to lower urinary pH, impaired renal function, and higher incidence rate of gout in men [29]. Separately, older age was found to be a predictor of declining urinary pH in stone-forming individuals, independent of decreasing kidney function and other urinary parameters reflecting dietary acid load [30].

Urinary pH is the major determinant of UA precipitation and stone formation [8,9,10]. At a urinary pH less than the dissociation constant (pKa of UA) of 5.35 at 37 °C, only about 96 mg/L of UA remains in solution. The solubility of UA increases sharply with increasing urinary pH [6]. In the present study, urinary pH increased significantly during the intake of the balanced mixed diet. However, low urinary pH remained the most common abnormality in the study population. A urinary pH below 5.8 was present in 85% of patients on their usual, self-selected diet and in 60% of patients on the standardized balanced diet. This finding confirmed that urinary pH is the predominant abnormality in UA nephrolithiasis.

Several etiologic factors are discussed with regard to acidic urinary pH. A higher dietary acid load from protein consumption has been found to lower urinary pH in healthy subjects [31,32]. In contrast, an ovo-lacto-vegetarian diet has been shown to increase urinary pH and alleviate the supersaturation of UA due to the strong alkalizing potential of fruits and vegetables [33]. Dietary protein intake has been reported to be higher in patients with UA urolithiasis than in non-stone forming subjects [20]. In the present study, urinary pH increased significantly under the balanced mixed diet, probably in response to the lower dietary acid load from animal protein intake, which is reflected by decreased urinary phosphate and sulfate excretion. However, mean urinary pH remained far below the therapeutic target value of 6.5 under the balanced mixed diet. These results suggest that lower urinary pH in UA nephrolithiasis can only be partially explained by diet.

The low urinary pH in UA stone patients has been observed for decades [34,35]. The acidic urinary pH was found to be due to a diet-independent acid load to the kidney and concomitant reduction in urinary buffering from impaired renal ammoniagenesis and/or excretion [9,10,36,37]. In the present study, urinary ammonium excretion did not differ significantly between the two diets, although animal protein intake was significantly lower under the balanced mixed diet. A comparison of the acid-base parameters of 38 patients with idiopathic UA stone disease with 38 matched control subjects under controlled diets revealed significantly lower urinary pH, higher net acid and titratable acid excretion, but similar 24 h urinary ammonium excretion in UA stone patients compared with the controls [38]. However, the exact underlying mechanisms of high net acid excretion in UA nephrolithiasis remain unclear [39].

Several features of the metabolic syndrome, including abdominal obesity, hyperglycemia, and insulin resistance, respectively, have been linked to low urinary pH [15,18,40,41,42]. A study of 148 non-stone forming individuals showed that urinary pH decreased with an increasing number of metabolic syndrome traits [41]. However, a recent study revealed that BMI was only weakly correlated with urinary pH, net acid excretion, and NH_4_^+^/net acid excretion among idiopathic UA stone patients, suggesting that factors other than obesity could contribute to UA stone formation [38]. Although the prevalence of overweight and obesity was extraordinarily high among the UA stone formers in the present study, no correlation was observed between urinary pH and body weight or BMI, respectively. Individually tailored approaches to weight reduction could reduce the risk of UA stone formation in patients with overweight and other components of the metabolic syndrome [43]. A randomized, controlled study on the impact of a conventional energy-restricted, lacto-vegetarian-oriented mixed diet with or without meal replacement on urinary risk factors for kidney stone formation and cardiometabolic risk profile in non-stone forming overweight women observed a higher relative weight loss, a higher rate of responders, and a significant decrease in serum UA concentration and the relative supersaturation of UA in the meal replacement group [44].

Low urine volume is a primary risk factor for UA stone formation, as the concentration and relative supersaturation of UA increases. In the present study, a urine volume of less than 2.0 L/24 h was diagnosed in 50% of patients on their usual diet. Although urine volume increased significantly with the balanced diet, 35% of the patients still had a urine volume of less than 2.0 L/24 h. It is suggested that the fluid intake with the standardized diet of 2.5 L/d did not compensate for the excessive sweating of the patients. Nutritional counselling should therefore ensure that fluid intake is adapted to individual requirements, which is achieved by the monitoring of urine volume by the patient. In the dietary treatment of UA stone formers, attention should be paid to a generous fluid intake, allowing a 24 h urine volume of at least 2.0 to 2.5 L. Since sufficiently high urine volume and urinary alkalization are essential therapeutic goals in UA urolithiasis, alkalizing beverages, especially bicarbonate-rich mineral waters and citrus juices, should be preferred for recurrence prevention in UA stone disease [7,23,45,46]. Therefore, self-monitoring of urinary pH is another important recommendation for the successful management of UA urolithiasis. A randomized trial in healthy individuals found that the intake of 2.0 L/d of a bicarbonate-rich mineral water resulted in a significant rise in urinary pH and, subsequently, a significant reduction in the risk of UA stone formation, computed as the relative supersaturation of UA, by approximately 70% [47]. This study also showed that the advantageous effects of mineral water rich in bicarbonate (1715 mg/L) on urinary pH and the relative supersaturation of UA were similar to those of oral sodium potassium citrate in equimolar alkali load [47].

Another urinary risk factor that predisposes to UA stone formation is hyperuricosuria. In addition to various genetic and environmental factors, acquired states of UA overproduction, such as a high dietary purine intake, may also contribute to hyperuricosuria [48]. Reduced animal protein intake is expected not only to increase urinary pH, but also to diminish UA excretion by lowering dietary purine intake. In the present study, mean urinary UA excretion did not differ significantly between the two diets, although purine intake was lower with the balanced mixed diet. However, the prevalence of hyperuricosuria in UA stone patients declined from 40% on the usual diet to 15% on the balanced mixed diet. Notably, previous studies revealed no difference in UA excretion between UA stone formers and normal control subjects, either on a free or fixed diet [10,38,49].

Hyperoxaluria and hypercalciuria are major risk factors for calcium oxalate stone formation. Although 85% of the study population had pure UA stones, hyperoxaluria was present in 25% of patients on the usual diet. Urinary oxalate excretion decreased significantly, and hyperoxaluria could no longer be diagnosed under the balanced diet. The decrease in urinary oxalate excretion was probably due to restriction of dietary oxalate intake. Hypercalciuria was present in 40% of patients on their usual diet but in only 15% of patients on the balanced mixed diet. Several dietary factors are known to affect urinary calcium excretion, particularly the dietary intake of calcium, animal protein, and sodium chloride [23]. Since the calcium content of both diets was similar, it is assumed that the significant decrease in urinary calcium excretion was rather due to the lower intake of sodium chloride and animal protein on the balanced diet. Hypercalciuria and hyperoxaluria are of particular concern in UA nephrolithiasis because UA may promote the formation of UA and calcium oxalate mixed stones through heterogeneous nucleation and epitaxial crystal growth [12,13,14]. A previous analysis of urinary stone composition revealed that about one-third of UA stones were mixed with calcium oxalate, whereas the remainder were pure [2].

The determination of the urinary risk profile on the usual diet revealed a high risk of UA and calcium oxalate stone formation in UA stone patients. Although urinary UA excretion did not change, the relative supersaturation of UA decreased significantly by 47% under the balanced diet, which was primarily due to the significant increase in urinary pH and volume. Moreover, the relative supersaturation of calcium oxalate declined significantly and to the same extent due to a significant decrease in urinary oxalate and calcium excretion and a significant increase in urine volume under the balanced diet. Estimates of the ion activity products of UA and calcium oxalate confirmed these results. The findings of the present study indicate that urinary calcium and oxalate excretion should also be monitored in patients with pure UA stones to reduce the risk of mixed stone formation with calcium oxalate. Dietary intervention was found to be a key component in the treatment of UA urolithiasis. Due to the alkalizing potential of fruits and vegetables, a lacto-vegetarian diet could be an option to support urinary alkalization. Further studies under standardized conditions are needed to evaluate the effects of a lacto-vegetarian diet on the risk of stone formation in UA nephrolithiasis.

## 5. Conclusions

To our knowledge, this is the first study to evaluate the effect of dietary intervention under controlled, standardized conditions on the urinary risk profile of UA stone patients. The findings of this study suggest that dietary intervention favorably affects urinary risk factors for UA stone formation, making it an effective treatment strategy for the management of UA nephrolithiasis. Urinary calcium and oxalate excretion should also be monitored in UA patients with pure UA stones to reduce the risk of mixed stone formation with calcium oxalate. Based on the results of this study, a lower urinary pH in UA stone patients can only be partially explained by diet. Although the role of overweight and obesity in UA nephrolithiasis remains unclear, weight reduction is recommended as an additional therapeutic approach to further reduce the risk of UA stone formation.

## Figures and Tables

**Table 1:** Characteristics of uric acid stone patients.

	Mean ± SD *n* (%)
Number of patients	20
Gender (men/women)	17/3
Age (years)	55.2 ± 12.8
BMI (kg/m^2^) ^a^	29.9 ± 3.7
BMI 18.5–24.9 kg/m^2 a^	2/19 (11%)
BMI 25.0–29.9 kg/m^2 a^	9/19 (47%)
BMI 30.0–34.9 kg/m^2 a^	8/19 (42%)
Systolic BP (mm Hg)	124 ± 12
Diastolic BP (mm Hg)	76 ± 5
[^13^C_2_]oxalate absorption (%) ^b^	6.8 ± 2.8
Type 2 diabetes	4/20 (20%)
Hypertension	10/20 (50%)
Hyperuricemia (*n*) ^c^	8/20 (40%)
Family history of stones (*n*)	5/15 (33%)

Abbreviations: BMI, body mass index; BP, blood pressure. ^a^ Total *n* = 19, because of missing height and weight data in 1 male patient. ^b^ Total *n* = 9, because of missing value in 8 male and 3 female patients. ^c^ Defined as serum uric acid >6.4 mg/dL.

**Table 2:** Urinary parameters under the usual diet and balanced mixed diet.

	Usual Diet *n* = 20 Mean ± SD	Balanced Diet *n* = 20 Mean ± SD	*p* Value
Volume (L/24 h)	1.884 ± 0.736	2.322 ± 0.803	0.015
Urinary pH	5.53 ± 0.40	5.87 ± 0.46	0.003
Density (g/cm^3^)	1.013 ± 0.005	1.006 ± 0.004	<0.001
Sodium (mmol/24 h)	188 ± 86	99 ± 40	<0.001
Potassium (mmol/24 h)	57 ± 22	58 ± 22	0.834
Calcium (mmol/24 h)	5.00 ± 3.00	3.28 ± 1.54	0.005
Magnesium (mmol/24 h)	4.58 ± 2.61	4.95 ± 2.14	0.199
Ammonium (mmol/24 h)	26.8 ± 13.0	21.4 ± 10.7	0.121
Chloride (mmol/24 h)	192 ± 87	103 ± 39	<0.001
Phosphate (mmol/24 h)	33.9 ± 11.7	27.2 ± 6.6	0.016
Sulfate (mmol/24 h)	24.5 ± 8.3	17.8 ± 3.2	<0.001
Creatinine (mmol/24 h)	16.2 ± 4.6	14.5 ± 3.3	0.058
Uric acid (mmol/24 h)	3.62 ± 1.22	3.22 ± 0.86	0.177
Oxalate (mmol/24 h)	0.415 ± 0.137	0.310 ± 0.079	0.001
Citrate (mmol/24 h)	3.016 ± 2.478	3.513 ± 1.943	0.165
AP Brushite index	3.15 ± 3.79	1.73 ± 2.53	0.006
AP Struvite index	0.67 ± 1.73	1.82 ± 5.28	0.105
AP Calcium oxalate index	1.26 ± 0.81	0.54 ± 0.42	<0.001
AP Uric acid (×10^−9^)	2.48 ± 1.49	1.24 ± 0.98	0.001
RS Brushite	0.461 ± 0.507	0.371 ± 0.489	0.202
RS Struvite	0.011 ± 0.024	0.028 ± 0.085	0.349
RS Calcium oxalate	6.236 ± 4.214	3.309 ± 2.918	<0.001
RS Uric acid	3.435 ± 1.958	1.821 ± 1.362	0.001

Abbreviations: AP, ion activity product; RS, relative supersaturation; SD, standard deviation.

**Table 3:** Urinary abnormalities under the usual diet and balanced mixed diet.

	Reference Range	Usual Diet *n* (%)	Balanced Diet *n* (%)
Volume (L/24 h)	<2.000	10 (50%)	7 (35%)
	≥2.000	10 (50%)	13 (65%)
Urinary pH	<5.40	8 (40%)	2 (10%)
	5.40–5.79	9 (45%)	10 (50%)
	≥5.80	3 (15%)	8 (40%)
Uric acid (mmol/24 h)	<4.0	12 (60%)	17 (85%)
	≥4.0	8 (40%)	3 (15%)
Calcium (mmol/24 h)	<5.0	12 (60%)	17 (85%)
	5.0–7.9	5 (25%)	3 (15%)
	≥8.0	3 (15%)	–
Oxalate (mmol/24 h)	<0.500	15 (75%)	20 (100%)
	≥0.500	5 (25%)	–
Citrate (mmol/24 h)	<2.500	10 (50%)	6 (30%)
	≥2.500	10 (50%)	14 (70%)

**Table 4:** Nutrient intake under the usual diet and balanced mixed diet.

	Usual Diet *n* = 20 Mean ± SD	Balanced Diet *n* = 20 Mean	*p* Value
Energy (kcal/day)	2391 ± 516	2355	0.952
Protein (g/day)	101 ± 25	71	0.001
Carbohydrates (g/day)	242 ± 66	327	0.001
Fat (g/day)	102 ± 28	81	0.017
Methionine (mg/day)	2199 ± 585	1415	<0.001
Cystine (mg/day)	1311 ± 337	835	<0.001
Cholesterol (mg/day)	459 ± 156	195	<0.001
Fiber (g/day)	20.3 ± 5.5	31.0	<0.001
Sodium (mg/day) ^a^	4337 ± 1821	2300	0.001
Potassium (mg/day)	2867 ± 536	3390	0.007
Calcium (mg/day)	974 ± 239	977	0.952
Oxalate (mg/day)	146 ± 45	121	0.078
Purines (mg/day)	579 ± 147	449	0.005
Alcohol (g/day)	11.3 ± 20.4	0	0.001
Water (mL/day)	2912 ± 837	3437	0.020

^a^ Estimated from urinary sodium excretion.

## Data Availability

The data are available upon reasonable personal request.
